# Glucosamine-Modified Reduction-Responsive Polymeric Micelles for Liver Cancer Therapy

**DOI:** 10.3390/molecules28093824

**Published:** 2023-04-30

**Authors:** Lei Meng, Fangshu Liu, Chenchen Du, Jiaying Zhu, Qian Xiong, Jing Li, Weitong Sun

**Affiliations:** College of Pharmacy, Jiamusi University, Jiamusi 154007, China

**Keywords:** liver cancer, glucosamine, sorafenib, reduction-responsive, polymer micelles

## Abstract

In this work, glucose transporter-1 (GLUT-1) and glutathione (GSH) over-expression in liver cancer was utilized to design a reduction-responsive and active targeting drug delivery system AG-PEG-SS-PCL (APSP) for the delivery of sorafenib (SF). The SF-APSP micelles were prepared using the thin film hydration method and characterized by various techniques. In vitro release experiments showed that the cumulative release of SF-APSP micelles in the simulated tumor microenvironment (pH 7.4 with GSH) reached 94.76 ± 1.78% at 48 h, while it was only 20.32 ± 1.67% in the normal physiological environment (pH 7.4 without GSH). The in vitro study revealed that glucosamine (AG) enhanced the antitumor effects of SF, and SF-APSP micelles inhibited proliferation by targeting HepG2 cells and suppressing cyclin D1 expression. The in vivo antitumor efficacy study further confirmed that the SF-APSP micelles had excellent antitumor effects and better tolerance against nude mouse with HepG2 cells than other treatment groups. All in all, these results indicated that SF-APSP micelles could be a promising drug delivery system for anti-hepatoma treatment.

## 1. Introduction

Liver cancer has remained one of the most lethal malignancies across the world for decades. It has emerged as the fourth most common cause of cancer-related mortality [1,2]. Sorafenib (SF) is the first FDA-approved molecular targeting drug for use in patients with unresectable liver cancer [3,4]. Nevertheless, the therapeutic efficacy of SF has dramatically decreased due to the presence of multidrug resistance (MDR). In addition, there are side effects such as poor aqueous solubility, low bioavailability, and cardiotoxicity, which seriously affect the clinical therapeutic effects of SF in cancer treatment [5,6]. To overcome these limitations, nano drug delivery system has emerged—for example, polymer micelles, nanoparticles, and liposomes [7,8]. Among these carriers, polymeric micelles have recently received considerable attention as effective nanocarriers. Polymeric micelles consist of amphiphilic copolymers with hydrophilic polar and hydrophobic nonpolar termini. The micelles have a core–shell structure, which consists of a hydrophobic core that can accommodate hydrophobic drugs and an outer shell comprising hydrophilic polymers which assist in warding off recognition via the immune system and extending blood vessel circulation [9]. Additionally, micelles have the capacity to target extraordinary tissues due to various targeting moieties which can be embellished on the surface of this system. Polymeric micelles exhibit a series of unique advantages as drug carriers, which can improve drug solubility, stability, and bioavailability, prevent drug degradation, reduce toxic effects, control drug release, and achieve specific targeting [10,11]. Hence, polymer micelles with all these benefits can be an excellent choice for carrying chemotherapeutics.

Currently, ligands and receptors are extensively used in targeted drug delivery. Tumor cells typically overexpress specific receptors on their cell membranes, which are potential targets for ligand selection [12,13,14,15,16]. The over-expression of the glucose transporter 1 (GLUT1), which has been validated in many cancer cells, including liver cancer cells, has been a target for drug therapy [17,18,19]. Glucosamine (AG), a glucose analogue, is recognized and transported into cells by GLUT1 on the tumor cell membrane, thereby increasing the intratumor accumulation of chemotherapeutics via receptor-mediated internalization. Many research studies have proven that AG exhibits anticancer activity via impact on biological pathways involved in cell death, apoptosis, cell proliferation, and angiogenesis [20,21,22,23]. Furthermore, AG can decrease the toxicity of drugs, specifically cardiotoxicity, in a novel polymer–drug conjugate [24]. Thus, AG is the ideal targeting ligand that has the capacity to modify micelles for tumor targeting, tumor inhibition, and reversal of drug resistance.

Apart from high stability and tumor targeting, an ideal drug delivery vehicle ought to exactly control the release of drug as well. In biological systems, there is a substantial distinction between the normal physiological environment and the tumor extracellular circumstances [25,26]. It has been indicated that the glutathione (GSH) level in cancerous cells is up to 1–11 mM, which is considerably higher than that in normal cells as well as in the extracellular fluid and blood. Therefore, a relatively strong reducing environment in tumor cells could be a promising biosignal for smart drug delivery [27,28,29,30].

**Figure 1:**
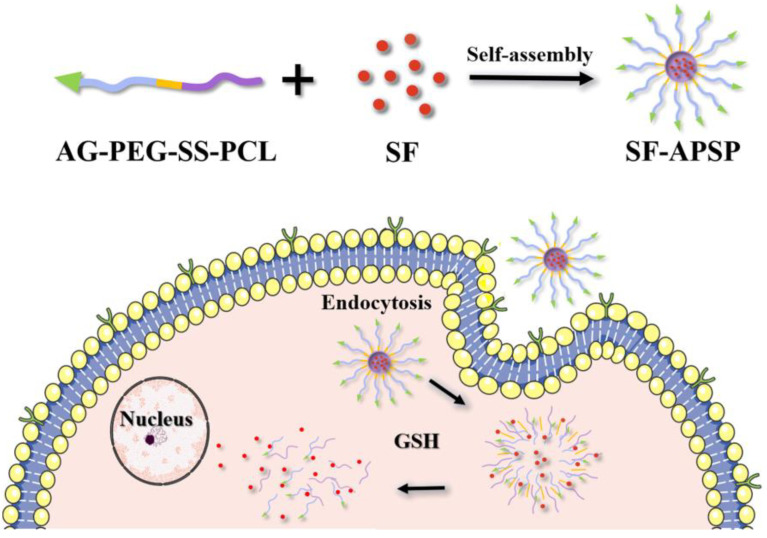
Scheme of drug entrapping and GSH-dependent release from polymeric micelles.

Consequently, in this study, the amphiphilic copolymer APSP was synthesized to deliver SF to the tumor site (Figure 1). In this nanoplat form, polyethylene glycol (PEG) and poly-ε-caprolactone (PCL) were used as the hydrophilic and hydrophobic segments, respectively, due to their favorable biocompatibility, biodegradability, and easy functional modification [31,32]. Disulfide bonds (-SS-) can be decomposed into thiols in the presence of GSH, so they were introduced to react to the high levels of GSH to allow rapid intracellular drug release. In addition, AG, a liver cancer-targeting ligand with anticancer effects, was also chosen to modify the polymer for tumor targeting. The SF-APSP micelles were designed for specific tumor cells with reduction-responsive characteristics, which allows the targeting of tumor cells through GLUT-1 and the modulation of drug release by GSH. The synthesis of the polymer, the preparation and characterization of the SF-APSP micelles, as well as the in vitro and in vivo assessments are elaborated in this paper.

## 2. Results and Discussion

### 2.1. Synthesis and Characterization of APSP

#### 2.1.1. Proton Nuclear Magnetic Resonance (^1^H NMR) Analysis

**Figure 2:**
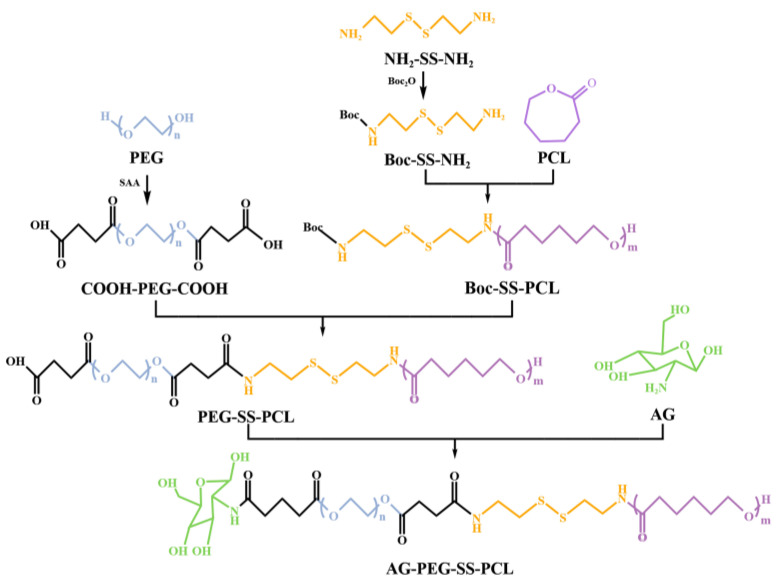
Synthesis route of APSP.

**Figure 3:**
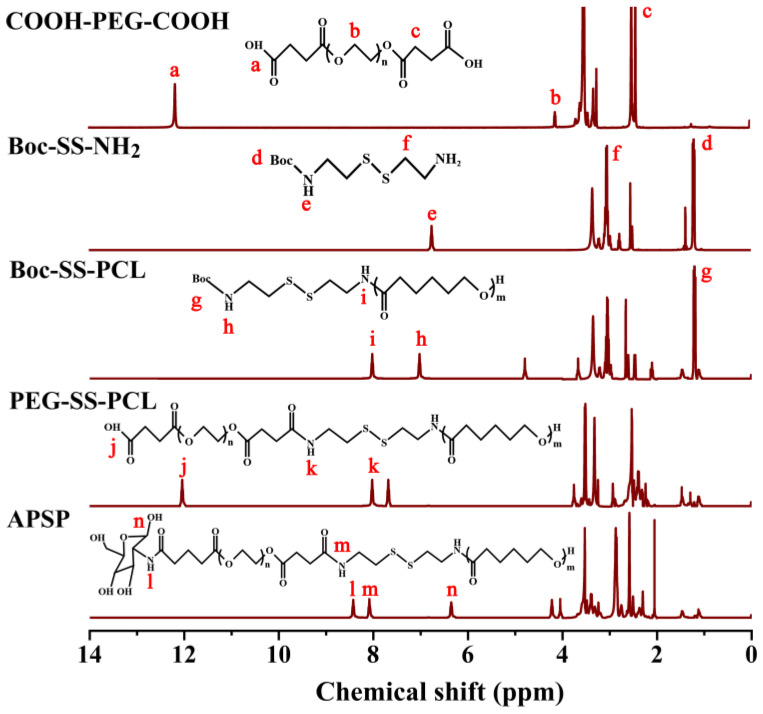
^1^H NMR spectra of COOH-PEG-COOH, Boc-SS-NH_2_, Boc-SS-PCL, PEG-SS-PCL, and APSP.

The synthesis route of APSP was shown in Figure 2. The chemical structure of APSP polymers was confirmed by ^1^H NMR spectroscopy (Figure 3). For COOH-PEG-COOH, signals at 2.46 ppm were attributed to the methyl peak of -COO- and -OC-. The peaks at 3.52 and 3.74 ppm belonged to the methylene peaks of -O-CH_2_- in the segment of PEG. In addition, there was a characteristic peak of the carboxyl group at approximately 12.22 ppm. The above results proved that the terminal dicarboxyl modification of the PEG was 1synthesized successfully. In the spectra of Boc-SS-NH_2_, the characteristic peaks at 1.19 ppm, 1.42 ppm, and 2.55~3.99 ppm were ascribed to (CH_3_)_3_-, (-CH_2_-NH_2_), (-S-CH_2_-CH_2_-) of the cysteamine dihydrochloride units, respectively. The presence of the characteristic peak of the amide bond at 6.77 ppm (-CO-NH-) in the spectrum, proved that di-tert-butyl dicarbonate was successfully connected to the cysteamine dihydrochloride. Compared with Boc-SS-NH_2_, several new signals were identified in the spectra of Boc-SS-PCL. The signals at 4.06 ppm, 2.29 ppm, 1.64 ppm, and 1.36 ppm belonged to the protons of the PCL segment, and the peaks at 8.04 ppm and 7.06 ppm were due to a new amide bond formation between the amino group of PCL and carboxyl group of Boc-SS-NH_2_. In the spectrum of PEG-SS-PCL, the characteristic peak of the carboxyl group appeared at 12.18 ppm, which indicated the formation of PEG-SS-PCL. Compared with the spectra of PEG-SS-PCL, the peak for the carboxyl group at 12.18 ppm disappeared in the ^1^H NMR spectra of APSP, replaced by the formation of a new amide bond at 8.14 ppm, which was significant evidence for the synthesis of APSP. These results confirmed that APSP conjugates were successfully synthesized.

#### 2.1.2. Fourier-Transform Infrared (FT-IR) Spectroscopy Analysis

**Figure 4:**
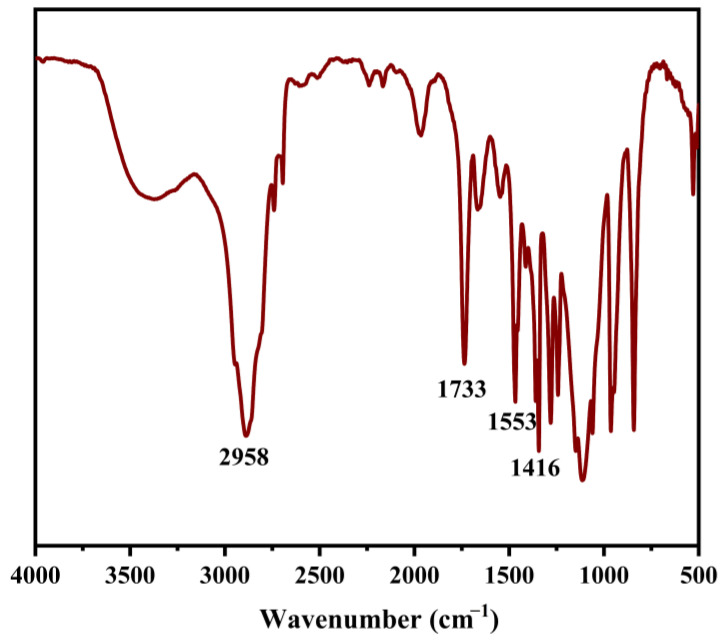
FTIR spectra of APSP.

To further demonstrate the polymer formation, the FTIR spectra of APSP are presented in Figure 4. The characteristic peaks at 2958 cm^−1^ appeared which corresponded to the N-H stretching vibrations in an amide bond, and the C=O in a carbonyl group. Moreover, there were distinct peaks at 1733 cm^−1^, 1553 cm^−1^, and 1416 cm^−1^, which were assigned to the C=O stretching vibration (amide I band), N-H bending vibration (amide II band), and C-N stretching vibration (amide III band) of the amide link, respectively. The above results indicated the successful synthesis of APSP. 

### 2.2. Critical Aggregation Concentration (CAC) Determination

**Figure 5:**
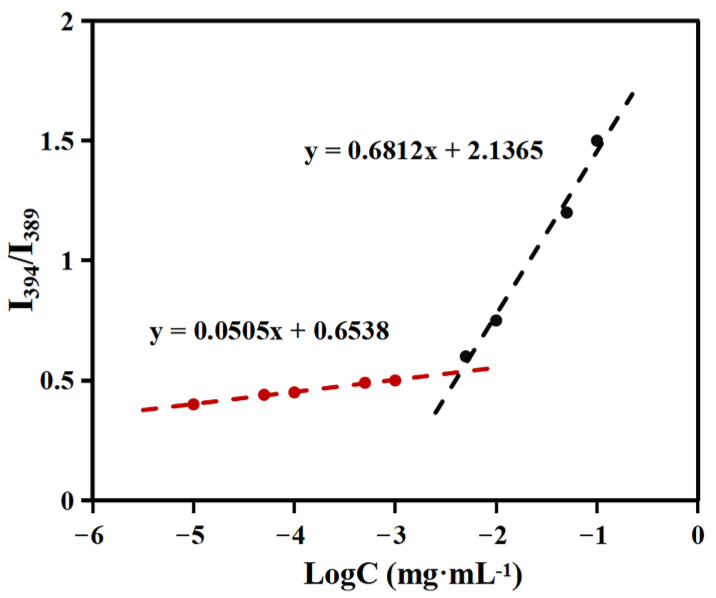
Plot of the fluorescence intensity ratio (I_394_/I_389_) against the logarithm of APSP concentration.

As shown in Figure 5, the value of I_394_/I_389_ ratio varied with increasing polymer concentration. These results demonstrated that pyrene molecules were transferred from a aqueous environment to a hydrophobic micellar microdomain. From the cross point of the two straight lines, the CAC of APSP was determined as 4.6 × 10^−3^ mg/mL, which suggested that APSP micelles would show excellent colloidal stability under dilute conditions.

### 2.3. Preparation and Characterization of SF-APSP Micelles

In this work, APSP copolymers were used as a micellar carrier for SF-loading and prepared using the thin film hydration method. According to the calculation method mentioned above, the DL% of SF-APSP micelles reached up to 8.44 ± 0.012%, and the EE% was 92.18 ± 0.15%.

**Figure 6:**
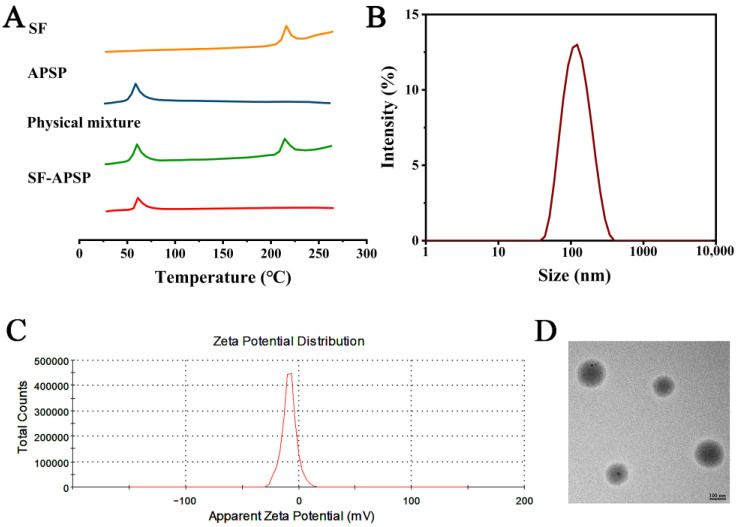
DSC profiles of SF-APSP micelles (**A**). Dynamic light scattering particle size (**B**) and zeta potential (**C**). Transmission electron micrograph of SF-APSP micelles (**D**).

The DSC spectra of SF, blank APSP micelles, physical mixture, and the SF-APSP micelles are shown in Figure 6A. SF had a sharp endothermic peak at 235 °C, and the melting temperature of the blank APSP micelles was 89 °C. For the physical mixture, the typical peaks of SF were still present in the thermogram. In contrast, the sharp endothermic peak of SF disappeared in the thermogram of the freeze-dried SF-APSP micelle powder, which indicated that SF was completely trapped in the hydrophobic core in the amorphous state, rather than adhering to the surface of the micelles.

The size and distribution, and zeta potential of SF-APSP micelles were measured by DLS in aqueous media (Figure 6B,C). The assay showed that the particle sizes of SF-APSP micelles showed a single distribution at the nanoscale. The mean size of SF-APSP micelles was 195.21 ± 3.32 nm and the poly-dispersity was observed as 0.14 ± 0.09. Furthermore, the zeta potential of SF-APSP micelles was −26.97 ± 0.06 mV. 

The morphological characterization of SF-PASP micelles was performed using TEM (Figure 6D). The micelle particles were spherical with smooth surfaces and were discrete from each other. The scale bar of the image shows the size of the micelles. 

### 2.4. Stability of Micelles 

**Figure 7:**
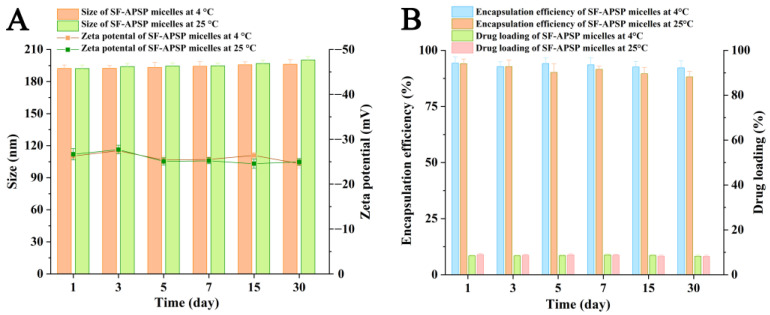
Particle size distribution and zeta potential of SF-APSP micelles (**A**). DL% and EE% of the SF-APSP micelles (**B**).

The stability of the micelles was evaluated by monitoring changes in particle size and zeta potential. As shown in Figure 7A, the mean size and zeta potential of micelles did not markedly change when stored at 4 or 25 °C for 1, 3, 5, 7, 15, or 30 days. Moreover, there were no significant variations in DL% and EE% during the storage time (Figure 7B), indicating that the micelles possessed excellent storage stability. 

### 2.5. GSH-Triggered Disassembly and Drug Release

**Figure 8:**
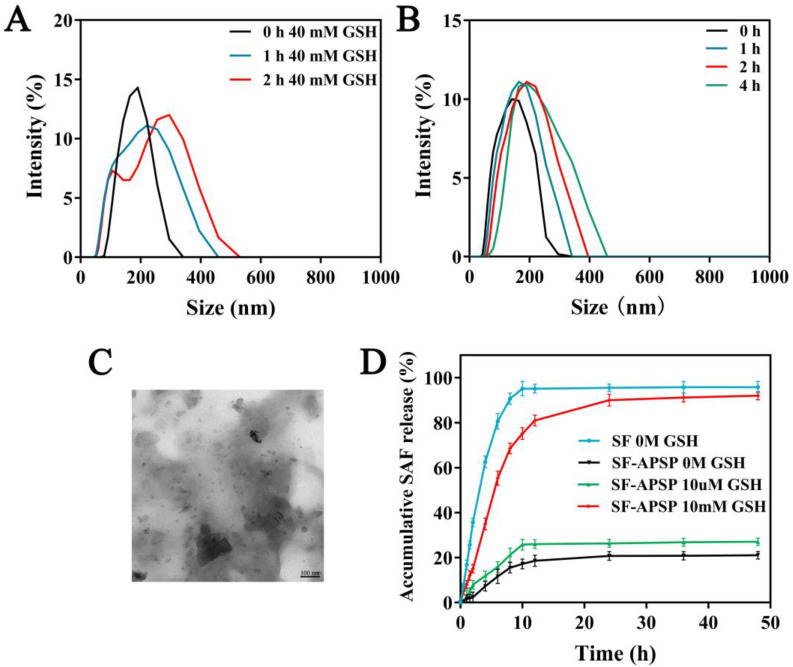
Size distributions of SF-APSP micelles at different time points in 40 mM GSH (**A**) and without GSH treatment (**B**). TEM images of SF-APSP micelles in 40 mM GSH for 4 h (**C**). Drug release profiles of SF-APSP micelles at different GSH concentrations (0 M, 10 μM, and 10 mM) at 37 °C (**D**).

The reduction-sensitivity of SF-APSP micelles was assessed by monitoring the time-dependent variation in micelle sizes in response to GSH (Figure 8A,B). The results from DLS showed that the size of SF-APSP micelles did not change significantly after incubation in the absence of GSH. In contrast, the size of SF-APSP micelles increased significantly with time in the presence of GSH. These morphological observations were in agreement with the DLS results (Figure 8C). These results demonstrated that SF-APSP micelles were highly sensitive to GSH.

In order to further investigate the reduction-sensitivity drug release behavior of SF-APSP micelles, PBS (pH 7.4) with different GSH concentrations was selected to simulate the environment of tumor tissues (Figure 8D). First, the free SF solution could be entirely released in about 8 h, while encapsulating SF in APSP could significantly delay the release rate of SF. Second, there was no clear burst of SF in SF-APSP micelles in GSH-free conditions. SF-APSP micelles showed relatively stable release properties, with only about 20.32 ± 1.67% of SF being released within 48 h, which indicated that the micelles were structurally intact under physiological conditions. Lastly, following incubation with 10 μM and 10 mM GSH for 48 h, the cumulative release of SF from SF-APSP micelles was 26.8 ± 1.05% and 94.76 ± 1.78%, respectively. This result further proved the high sensitivity of SF-APSP micelles to GSH.

### 2.6. In Vitro Cellular Uptake 

**Figure 9:**
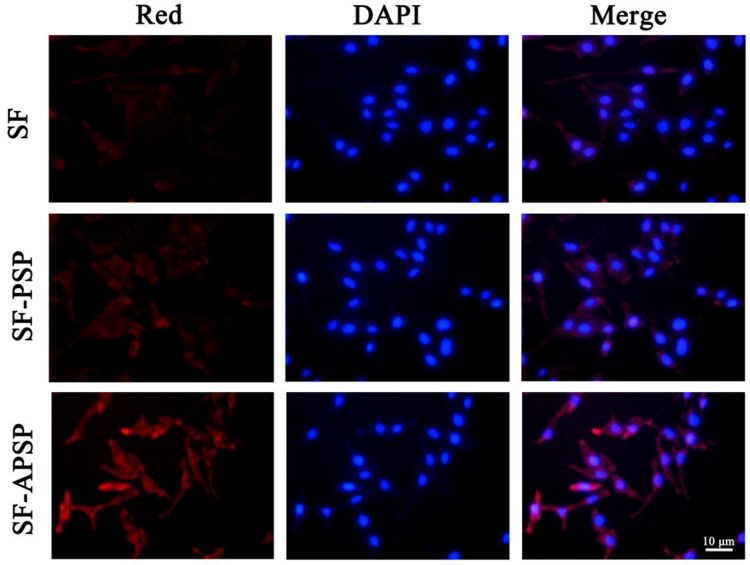
Cellular uptake of different SF formulations by HepG2 cells after incubation for 8 h (blue and red represented the cell nucleus and SF, respectively).

The red fluorescence of Nile red was obvious in the SF-APSP micelles group, while the free Nile red and SF-PSP micelles groups exhibited weak fluorescence. The Nile red released from SF-APSP micelles was stronger than SF-PSP micelles, indicating that the SF-APSP micelles had a significant active targeting effect and could be taken up effectively by the HepG2 cells (Figure 9). Modification of the vector by AG enhanced the binding ability of the micelles to AG-positive cells through AG receptor-mediated targeted delivery, which could be demonstrated by the highest fluorescence accumulation in HepG2 cells of the SF-APSP micelles group. 

### 2.7. In Vitro Cytotoxicity Study 

**Figure 10:**
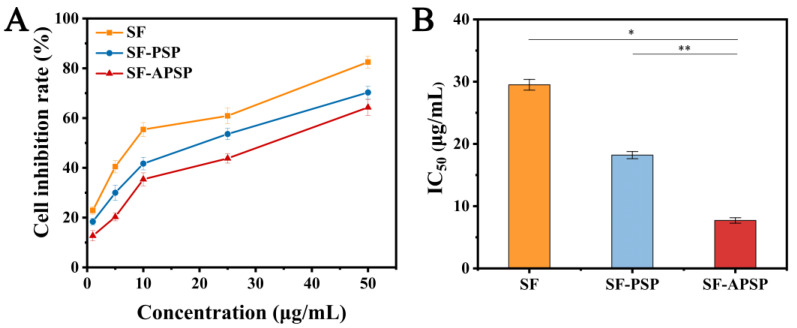
Cell inhibition rate (**A**) and IC_50_ values (**B**) after incubating with different SF formulations for 48 h (* *p* < 0.05, ** *p* < 0.01).

For all preparations, the cell viability decreased in the presence of increasing SF concentrations, suggesting the concentration-dependent uptake of both the SF solution and SF-loaded micellar preparations by HepG2 cells (Figure 10A). The IC_50_ values of the SF solution, SF-APSP micelles, and SF-PSP micelles were 29.5, 18.2, and 7.7 μg/mL, respectively (Figure 10B). The SF-loaded micellar preparations exhibited remarkable cytotoxicity, indicating that the designed reduction-sensitive conjugate could boost the inhibitory effect of SF on HepG2 cells. Compared with the SF-PSP micelles, the AG-linked SF-APSP micelles exhibited the highest cytotoxicity towards HepG2 cells at equal SF dosages. 

The above results indicated that SF-APSP micelles had excellent antitumor potential. The reduction-sensitive effect of the SF-APSP micelles and the targeting capability of AG increased the accumulation of SF in tumor cells. AG modification on the surface of the micelle greatly promoted its specific uptake by HepG2 cells through GLUT protein-mediated endocytosis. Meanwhile, AG exerted its anticancer activity by targeting multiple signaling pathways and genes involved in tumor development [33]. The combination therapy of AG and SF might effectively inhibit tumor cells and enhance the antitumor effect of SF.

### 2.8. In Vitro Wound Healing Assay

**Figure 11:**
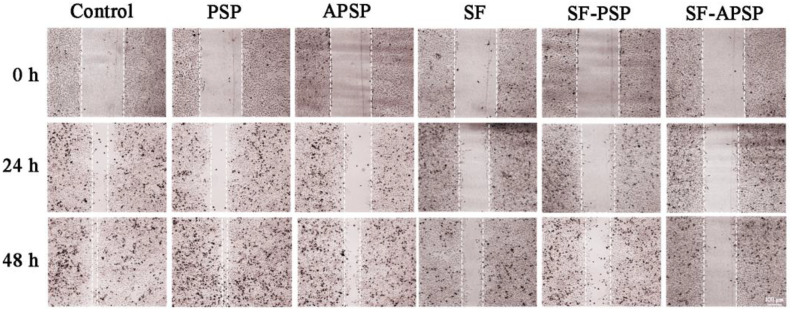
Inhibition of HepG2 cell migration after incubation with different preparations for 48 h (SF concentration: 50 pg/mL).

The inhibition of HepG2 cell migration by SF-APSP micelles was investigated using the wound healing assay. The artificial scratches healed after 24 and 48 h of incubation for the untreated control HepG2 cells(Figure 11). The migration potential of HepG2 cells was significantly inhibited by 50 pg/mL SF-APSP micelles at 24 and 48 h in comparison with the other groups, which is critical for prevention of tumor metastasis. 

### 2.9. Western Blot Analysis

**Figure 12:**
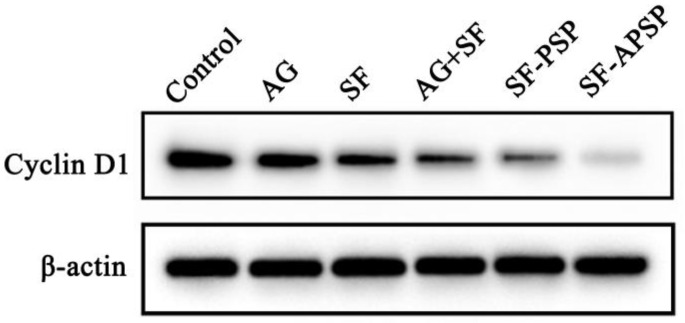
Protein expression levels of cyclin D1 measured by Western blot analysis after 12 h of incubation with different preparations (SF concentration: 10 μM/mL).

Cyclin D1 is a key factor in cell proliferation in many forms of cancers [34]. The Western blot assay showed that the protein levels of cyclin D1 were significantly downregulated in all other groups compared to the control (Figure 12). The expression of cyclin D1 in HepG2 cells decreased more when exposed to the combination treatment compared to AG or SF alone, suggesting that SF and AG could synergistically suppress cell proliferation. The expression of cyclin D1 protein was significantly decreased in the SF-APSP group compared to the SF-PSP group. This suggests that AG was able to enhance the anti-proliferative effects of SF in HepG2 cells, which might be due to its dual functional properties as an antitumor and targeting ligand. 

### 2.10. In Vivo Therapeutic Efficacy

**Figure 13:**
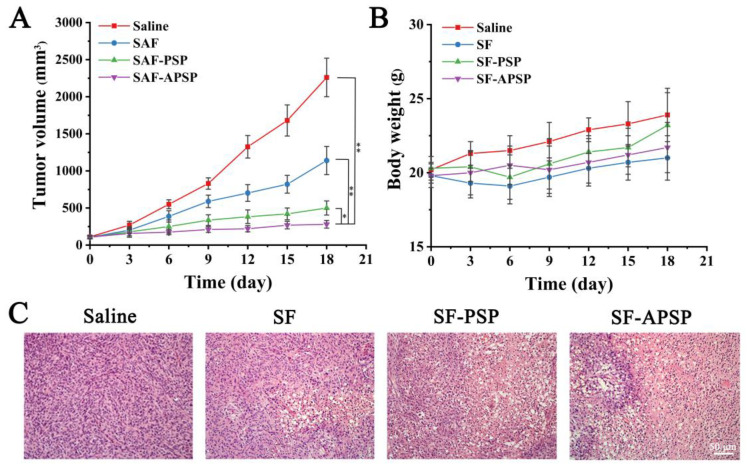
In vivo antitumor activity of different SF formulations (20 mg SF/kg) in a human liver cancer xenograft model (HepG2) with saline as control. Tumor growth curves (**A**) and body weight curves (**B**) of each group of tumor-bearing mice (*n* = 5) after treatment with different preparations given by intravenous injection. H&E-stained sections of tumor tissues (**C**) Data are shown as mean ± SD (* *p* < 0.05, ** *p* < 0.01).

The generated tumor growth curves obtained by measuring the tumor volume after treatment are shown in Figure 13A. The tumors treated with saline could not be controlled and grew quickly. The tumor treated with free SF grew progressively, but at a slower rate than that of the control group. The tumor volumes in the SF-PSP micelles and SF-APSP micelles groups were markedly smaller than that in all other groups after treatments (*p* < 0.05). Importantly, mice treated with AG-modified drug carriers showed the strongest tumor inhibition effect and significantly reduced tumor development. There was no discernible difference between the micelle and saline groups, and the body weight of the free SF group was lower than that of the saline group (Figure 13B).

The histopathological results are shown in Figure 13C. The normal saline group showed dense tumor cells, a fully developed nucleus, and basically no apoptosis or necrosis of tumor cells. In contrast, the morphology of the tumor tissue varied to variable degrees in the other treatment groups. Notably, tissue sections from animals undergoing therapy with SF-APSP micelles showed the lowest number of cancer cells while exhibiting the highest level of apoptosis. Additionally, the apoptotic cells presented the representative feature of chromatin condensation or nuclei fragmentation, indicating that SF-APSP micelles had a stronger tumor inhibition effect. Such finding further verifies that AG modification caused the formulation to exhibit targeted and synergistic effects and improved the anticancer efficacy of the preparation in vivo. 

### 2.11. Discussion

The high expression of glucose transporters on the surface of liver cancer cells was exploited. The ligands that have selective and affinity for tumor cells were attached to the micelle surface, and the recognition of the ligands by specific receptors improved the targeting of the micelles, thus increasing the accumulation of drug-laden micelles at the liver cancer sites. Meanwhile, the introduction of disulfide-modified polymeric carriers that were sensitive to reducing GSH conditions increased the efficacy of the drug and reduced the adverse effects by inducing the micelles to release their cargo into the tumor cells in response to environmental stimulation. AG enhanced the antitumor effects of SF. With the synergistic effect of AG and SF, SF-APSP micelles inhibited proliferation by targeting HepG2 cells and suppressing cyclin D1 expression. SF-APSP micelles exhibited significant in vivo and in vitro antitumor effects. Taken together, these results demonstrate the importance of SF-APSP micelles as a prospective anticancer drug that promises to be a clinical treatment option for liver cancer.

## 3. Materials and Methods

### 3.1. Materials 

Sorafenib was purchased from Dalian Meilun Biotechnology Co., Ltd. (Dalian, China). Polyethylene glycol (Mw = 2000) and trifluoroacetic acid (TFA) were obtained from Sigma-Aldrich (Shanghai, China). Triethylamine, succinic anhydride, cystamine dihydrochloride (CSA), N,N-dicyclohexylcarbodiimide (DCC), N-hydroxysuccinimide (NHS), 1-(3-dimethylaminopropyl)-3-ethylcarbodiimide hydrochloride (EDC), pyrene, and reduced GSH (purity = 98%) were purchased from Aladdin Chemical Company, Ltd. (Shanghai, China). ε-caprolactone, stannous octoate, di-tert-butyl dicarbonate, sodium dodecyl sulphate (SDS), and glucosamine hydrochloride were purchased from Shanghai Macklin Biochemical Co., Ltd. (Shanghai, China). Fetal bovine serum (FBS), phosphate-buffered saline (PBS), 4,6-diamidino-2-phenylindole (DAPI), RPMI 1640 medium, trypsin, antibodies, and the CCK-8 cell kit were obtained from Beyotime Biological Technology Co., Ltd. (Beijing, China). All antibodies in this paper were all supplied from Biolegend. All other reagents were of analytical or chromatographic grade. 

### 3.2. Cells and Animals

The human hepatocellular liver carcinoma (HepG2) cells were supplied by the College of Basic Medicine, Jiamusi University (Jiamusi, China). After which, the cells were cultured in RPMI 1640 (Gibco, Carlsbad, CA, USA) medium with 1% penicillin-streptomycin and 10% FBS in a humid atmosphere with 5% CO_2_ at 37 °C.

BALB/c mice, aged 4 to 6 weeks old, were purchased from Liaoning Changsheng Biotechnology Co., Ltd. (Liaoning, China). These mice were fed under the same conditions. The in vivo experiments were performed according to the guidelines of the Experimental Animal Administrative Committee of Jiamusi University. 

### 3.3. Synthesis of APSP

In this study, APSP were prepared by reactions involving several steps. Firstly, polyethylene glycol (PEG) was attached with butanedioic anhydride to form double carboxyl terminated polyethylene glycol (COOH-PEG-COOH). Next, di-tert-butyl dicarbonate was used to protect the amine group at one end of the cysteamine dihydrochloride to obtain Boc-SS-NH_2_. Then, polycaprolactone was introduced to prepare Boc-SS-PCL by forming an amide bond between the primary amino groups of Boc-SS-NH_2_ and carboxyl groups of polycaprolactone, and then Boc deprotection was carried out using TFA to obtain NH_2_-SS-PCL. Then, PEG-SS-PCL (PSP) was obtained via the formation of the amide bond between the primary amino groups of NH_2_-SS-PCL and the carboxyl groups of COOH-PEG-COOH. Lastly, APSP was obtained by combining PEG-SS-PCL with glucosamine hydrochloride by carbodiimide chemistry.

#### 3.3.1. Synthesis of COOH-PEG-COOH

COOH-PEG-COOH was obtained by combining succinic anhydride (SAA) to PEG through an esterification reaction. Briefly, 2 g of PEG with a MW of 2000 g/mol and 0.3 g of SAA were mixed in anhydrous toluene and the reaction proceeded at 110 °C for 1.5 h. Afterwards, toluene was removed by rotary evaporation, and the resulting COOH-PEG-COOH was then purified by cold ether precipitation. 

#### 3.3.2. Synthesis of Boc-SS-NH_2_

To obtain Boc-SS-NH_2_, 1 g of cysteamine dihydrochloride was dissolved in 20 mL of methanol, and 2.85 mL triethylamine was added at RT. Subsequently, 0.7 g of di-tert-butyl dicarbonate, which had been dissolved in 5 mL of methanol, was added with stirring for 3 h. A hydrochloric acid solution (0.2 mol·mL^−1^) was added to the solution to remove unreacted di-tert-butyl decarbonate; 1 M NaOH was used to adjust the pH to 9, and then the solution was extracted with chloroform. At last, the methanol was removed under reduced pressure to obtain Boc-SS-NH_2_.

#### 3.3.3. Synthesis of Boc-SS-PCL

The obtained product Boc-NH-SS-NH_2_ (0.09 g) was dissolved in chloroform, before triethylamine (TEA) (4 mL) was added and mixed with poly (ε-caprolactone) under the catalysis of stannous octoate. After stirring at 80 °C for 24 h, the mixture was sedimented and purified with cold ether. Then, the deprotection of Boc-SS-PCL was performed by adding trifluoroacetic acid (TFA) and stirring in an ice bath for 15 min. The NH_2_-SS-PCL conjugates were purified by precipitation with cold ether.

#### 3.3.4. Synthesis of PEG-SS-PCL

One of the two carboxyl groups of COOH-PEG-COOH (1 g) was activated using DCC (0.1 g) and NHS (0.1 g) in dimethyl sulfoxide at RT for 5 h. Then, NH_2_-SS-PCL was dissolved into the above solution in the presence of triethylamine. The mixture was stirred for 48 h until the NH_2_-SS-PCL reacted completely. The resulting product of PEG-SS-PCL was purified by dialysis with deionized water and freeze-drying.

#### 3.3.5. Synthesis of APSP

The APSP was synthesized using the following method. Briefly, 0.217 g of PEG-SS-PCL was added to 20 mL deionized water and then the carboxyl groups of PEG-SS-PCL were activated by NHS and EDC for 5 h. A total of 0.215 g of glucosamine hydrochloride and triethylamine (TEA) were then added and the solution was stirred overnight at RT. The resulting APSP product was purified by dialysis against deionized water for 24 h and obtained after freeze-drying. All the structures were identified by ^1^H-nuclear magnetic resonance (^1^H NMR, Bruker AV600, Karlsruhe, Germany) and Fourier-transform infrared spectrophotometry (FTIR, PerkinElmer Spectrum100, Waltham, MA, USA) and spectroscopy.

### 3.4. Critical Aggregation Concentration (CAC) Determination

The critical aggregation concentration (CAC) of micelles was determined with pyrene as a fluorescent probe [35]. The self-assembly behavior of APSP in an aqueous environment can be predicted from its CAC value. To obtain sample solutions, the APSP solution was sonicated as described above and diluted to obtain the desired concentrations. A quantitative amount of pyrene in acetone was added to a series of 10 mL vials and the acetone was volatilized. Subsequently, the APSP solution was then added to the pyrene-containing vials along with deionized water in various volume ratios to obtain the desired concentrations of APSP, which were left to equilibrate under agitation for 24 h away from light. The excitation wavelength was 390 nm and the emissions were monitored at wavelengths ranging from 200 to 600 nm. The ratio of the first and third intensity peaks was plotted against the log concentration of the solution to determine the CAC.

### 3.5. Preparation and Characterization of SF-APSP Micelles

In this study, SF-APSP micelles were obtained using the thin film hydration method (TFHM). About 10 mg of APSP and 1 mg of SF were added to 5 mL of methanol, and then the organic solvent was evaporated to form a dry film. SF-APSP micelles were formed by dissolving the drug-loaded film in deionized water. Finally, the product was lyophilized to obtain a SF-APSP micelle powder. The blank micelles were produced using the same method without the addition of SF. 

The particle size, zeta potential, and polydispersity index (PDI) were determined by dynamic light scattering (DLS, Nano-s, Malvern Instruments Ltd., UK). Additionally, the morphology of the SF-APSP micelles was characterized using transmission electron microscopy (TEM, HT7700, Hitachi Ltd., Tokyo, Japan). The thermal behavior was characterized using differential scanning calorimetry (DSC, STA409PC, Hengjiu Ltd., Xuzhou, China). The drug loading (DL%) and encapsulation efficiency (EE%) of the SF-APSP micelles were determined using an HPLC system at 265 nm, and computed using the following formula:

DL% = (mass of SF in micelle/mass of total micelle) × 100%

EE% = (mass of SF in micelle/mass of SF added during preparation) × 100%.

### 3.6. Stability Study

For pharmaceutical formulations, their stability during storage is very important. In order to examine stability, SF-PASP micelles were stored in sealed glass vials at 4 and 25 °C for 1, 3, 5, 7, 15, and 30 days and then the particle size and zeta potential were measured and recorded. DL% and EE% were calculated using the above method.

### 3.7. GSH-Triggered Drug Release and Micelle Disassembly

In this work, the reduction sensitivity of SF-APSP micelles was monitored in PBS (pH 7.4) with or without GSH. Briefly, the SF-APSP micelles were transferred into the dialysis bags (MWCO = 3500 Da), which were submerged in release medium and then incubated in PBS (pH 7.4) on a thermostatic shaker with a rotational speed of 100 rpm at 37 °C, with different GSH concentrations (0 and 40 mM) for 4 h. These samples were then monitored by DLS for changes in size distribution at different time points, and the morphology of the micelles in 40 mM GSH conditions was also investigated by TEM.

The in vitro release of SF-APSP micelles was investigated by the dialysis method. Generally, the dialysis bag (MWCO: 3.5 kDa) containing the SF-APSP micelle solution was immersed in release media. PBS solutions containing 0.5% (*w/v*) sodium dodecyl sulfate (SDS) with different concentrations of GSH (0, 10 μM, and 10 mM) were used as the media at 37 °C with shaking at 100 rpm. During dialysis, 5 mL of the release medium were sampled by replacing with fresh medium at appropriate time intervals. The amount of SF released was then calculated using HPLC analysis. 

### 3.8. In Vitro Cellular Uptake

In order to facilitate the observation of cellular uptake, the hydrophobic fluorescent agent Nile red was used as a hydrophobic drug analogue and encapsulated into the micellar system to simulate SF. Red-loaded APSP and PSP micelles were prepared according to the preparation of SF-APSP micelles. In brief, HepG2 cells were inoculated into 6-well plates (1.5 × 10^5^/well) and cultured overnight. Afterwards, this medium was replaced by RPMI 1640 medium containing Nile red solution, Red-APSP micelles, and Red-PSP micelles. After incubated for 1.5 h, the cells were washed three times with PBS and immobilized with 2.5% paraformaldehyde. Then, the nuclei were stained with DAPI for 2 min in dark. Subsequently, the supernatant media was aspirated and washed 2 times with PBS. The uptake behavior was observed using an inverted fluorescence microscope (Leica, DMIL, Wetzlar, Germany).

### 3.9. Cell Cytotoxicity Assays

The in vitro cytotoxicity of free SF, APSP micelles, SF-APSP micelles, and SF-PEG-SS-PCL (SF-PSP) micelles was detected using CCK-8 assays in HepG2 cells. Briefly, HepG2 cells were added at 1 × 10^4^ cells per well into 96-well plates and incubated for 24 h at 37 °C. After fully attached, the culture medium was replaced with fresh medium containing SF solution, APSP micelles, SF-PSP micelles, SF-APSP micelles, or medium alone. Free drug was dissolved using DMSO and diluted to less than 0.1%. After 48 h incubation, fresh medium (100 μL) and CCK-8 (10 μL) were added to all wells separately and incubated successively for 4 h. Finally, the absorbency of each well at 450 nm was measured using a microplate reader. Cell viability was calculated using the following equation:

Cell viability% = [(As − Ac)/(Ab − Ac)] × 100%

where As, Ab, and Ac represent the absorbances of the treated group, control group, and the blank plate, respectively. 

### 3.10. Wound Healing Assay

The inhibition of HepG2 cells migration by the SF solution, SF-PSP micelles, and SF-APSP micelles was investigated using a wound healing assay. In brief, HepG2 cells were seeded into 6-well plates and grown to near 100% confluency. After that, a scratch was made within the cell layer using the tips of sterile pipettes followed by multiple washings with PBS. The scratched monolayer of HepG2 cells was treated with different formulations (SF solution, SF-PSP micelles, and SF- APSP micelles [50 pg/mL each] and blank APSP micelles). Images of different groups were captured at 0, 24, and 48 h using an inverted fluorescence microscope. Untreated cells were used as the control. 

### 3.11. Western Blot Analysis

HepG2 cells were cultured overnight under 5% CO_2_ at 37 °C. Then, 10 μM of AG solution, SF solution, and SF-loaded micelles were added, followed by a 12 h incubation. Then, total proteins were extracted from the HepG2 cells and protein concentrations were standardized using a BCA protein assay kit (Solarbio, Beijing, China). 

The target proteins were separated by SDS-polyacrylamide gel electrophoresis (SDS-PAGE) and transferred to polyvinylidene difluoride (PVDF) membranes. Next, the membranes were blocked with 5% skimmed milk powder, and then incubated with primary antibodies overnight at 4 °C. The signal was visible after incubation with a secondary antibody at RT for 1 h.

### 3.12. In Vivo Therapeutic Efficacy

To investigate the antitumor therapeutic effect of SF-APSP micelles, male BALB/c nude mice were subcutaneously inoculated with HepG2 cells at a density of 5 × 10^6^ cells/mouse. The HepG2 tumor-bearing mice were separated into four groups (*n* = 5) when the tumor volume was approximately 100 mm^3^. Subsequently, the mice were intravenously administered with saline, SF, SF-PSP micelles, and SF-APSP micelles every 3 days (20 mg SF/kg). Meanwhile, changes in tumor volume and weight of the mice were recorded regularly. 

After the in vivo therapeutic efficacy analysis was completed, the tumors were surgically excised, weighed, fixed, embedded in paraffin, and sectioned. The sections were stained with H&E to observe the apoptosis of tumor cells.

### 3.13. Statistical Analyses

The data for each experimental were analyzed using Graph Pad Prism 9.0 software. The results were presented as “mean ± SD” and one-way ANOVA was used for comparison between groups. The *p* values lower than 0.05 (*p* < 0.05) were regarded as statistically significant.

## 4. Conclusions

In summary, reduction-sensitive and hepatoma-targeting SF-APSP micelles were successfully fabricated in order to treat liver cancer. The SF-APSP micelles exhibited a GSH-dependent pattern of drug release. The SF-APSP micelles exhibited significant tumor suppressive effects, efficiently delivered SF to HepG2 cells through receptor-mediated endocytosis, and effectively inhibited the proliferation of cells by downregulating cyclin D1. Overall, the SF-loaded dual-function micelles more markedly inhibited the proliferation of HepG2 cells in vitro and in vivo. Hence, we believe that this new drug delivery system has a broad potential for effective liver cancer treatment.

## Data Availability

Data will be made available on request.

## Sample Availability

Samples of the compounds are not available from the authors.
